# Obfuscating encrypted threshold signature algorithm and its applications in cloud computing

**DOI:** 10.1371/journal.pone.0250259

**Published:** 2021-04-16

**Authors:** Yahong Li, Jianzhou Wei, Bin Wu, Chunli Wang, Caifen Wang, Yulei Zhang, Xiaodong Yang

**Affiliations:** 1 School of Electronic and Information Engineering, Lanzhou Jiaotong University, Lanzhou, Gansu, China; 2 School of Computer Science and Engineering, University of Electronic Science and Technology of China, Chengdu, Sichuan, China; 3 College of Science, Gansu Agricultural University, Lanzhou, Gansu, China; 4 College of Computer Science and Engineering, Northwest Normal University, Lanzhou, Gansu, China; 5 College of Big Data and Internet, Shenzhen Technology University, Shenzhen, China; Northeastern University at Qinhuangdao, CHINA

## Abstract

Current cloud computing causes serious restrictions to safeguarding users’ data privacy. Since users’ sensitive data is submitted in unencrypted forms to remote machines possessed and operated by untrusted service providers, users’ sensitive data may be leaked by service providers. Program obfuscation shows the unique advantages that it can provide for cloud computing. In this paper, we construct an encrypted threshold signature functionality, which can outsource the threshold signing rights of users to cloud server securely by applying obfuscation, while revealing no more sensitive information. The obfuscator is proven to satisfy the average case virtual black box property and existentially unforgeable under the decisional linear (DLIN) assumption and computational Diffie-Hellman (CDH) assumption in the standard model. Moreover, we implement our scheme using the Java pairing-based cryptography library on a laptop.

## Introduction

Cloud computing provides various data storage and services over a network [1]. Due to its many benefits, it collaborates with other promising technologies such as 5G networks [2, 3] and IoT [4, 5]. Meanwhile, more individual and corporate gradually outsource data storage or computation to the cloud for its cost saving and convenience. Despite various merits of cloud computing, however in practice, cloud servers are not entirely reliable [6–8]. Since if users directly delivery their data to cloud platforms, the important information in data will be leaked to cloud servers, which will lead to the exposure of users’ privacy. Therefore, the concern is how to secure the data and rely on the services in cloud.

Obfuscation and cryptography are powerful tools that protect the data of users from a malicious/curious cloud server while preserving the services [9, 10]. When user chooses the cloud service to finish computation task without knowing the sensitive information of the task. In this case, the user data are obfuscated before they are forwarded to the cloud server. In a word, the cloud server can finish the computation tasks without sacrificing data privacy [11]. The related researches have addressed various security and privacy issues on data outsourcing. The works in [12–14] proposed cloud storage system in which data were obfuscated and encrypted. Sugumar et al. [15] proposed a confidentiality system named as SUG-DO (SUGUMARDigits Obfuscation) to enhance the security of data in cloud environment. Jin et al. [6] proposed an attribute-based data sharing scheme especially for resource-constrained mobile users in cloud computing. Recently, some new privacy preserving schemes [16–18]for other features have been reported to meet different auditing requirements in the literature.

Threshold cryptography is an essential distributed computational paradigm for enhancing the applicability and the security of public key schemes [19]. This approach was based on the work of Shamir [20], who proposed the definition of (*t*, *n*) threshold secret sharing scheme. Using Shamir’s idea, (*t*, *n*)-threshold signature [21] separates private keys into *n* shares distributed to different users, *t* threshold or more share holders need cooperatively produce a signature. Most existing threshold algorithms rely on the trusted dealers (TDs) to distribute secrets, and more than that, it needs to be always trusted and safeguarded because it has the private key for all users, but they usually do not keep the confidentiality of the data against the cloud. To eliminate the third party, Pedersen [22] was the first to present threshold secret sharing scheme without any TDs. And then, based on the ideas similar to the protocol of Pedersen, Gennaro et al. [23] presented a secure distributed key generation for discrete log based cryptosystems (GJKR’s DKG protocol) that enjoyed a full proof of security. Due to its distributed nature and the lack of a central authority, threshold cryptography becomes one of the most important tools in offering secure applications such as password protection [24] and cloud computing [25]. These studies make great contributions for protecting security of information systems and against various attacks. However, for nontrustable cloud, secret cryptographic keys are potentially vulnerable to attackers, the problem is related to ensuring proper protection of the outsourced computation task.

Program obfuscation is a very hot research topic in the field of practical application points of view, since program obfuscators perfectly conceal important information encoded into programs. A major breakthrough arrived with the work of Barak et al. [26] put forward the concept of program obfuscation into the area of cryptography, their work showed that the construction of generic obfuscation was impossible under the virtual black-box property. Many other impossibility results have been demonstrated in many situations [27, 28]. However, there are a few positive results for some functions in [29, 30]. Faced with the applications of cryptographic functionality, the first ever obfuscated re-encryption was mentioned in TCC’2007 by Hohenberger [31], a new security concept of average case virtual black-box property (ACVBP) was proposed. Succeeding their groundwork, Hada [32] proposed an obfuscator for encrypted signature scheme, and extended the definition of ACVBP, the algorithm was secure under the decisional linear assumption in the standard model. Consequently, several different functionalities and the corresponding obfuscators have been proposed. The research [33] showed a type of obfuscator for verifiably encrypted signatures. The obfuscation of encrypted group signature (EGS) was studied in [34], where the notion of ACVBP w.r.t *R*(*C*) and *T*(*C*) was defined. To provide secure authentication, Yang et al. [35], applied an obfuscator to anonymous authentication, the algorithm supported batch verification of authentication requests, realizing the improvement of efficiency. In order to make obfuscation application into cloud computing, Zhang et al. [36] proposed an obfuscator for all polynomial-size CNF circuits and used to cloud computing. Zhang et al. [37] proposed an obfuscator for encrypted verifiable encrypted signature, and modelled the application in electronic transactions. The obfuscation can achieve a series of applications, threshold signature is an attractive service used in cloud computing, this paper focuses on achieving encrypted threshold signature, which designs an obfuscator to protect users’ privacy. It should offer outsourcing computation without compromising data privacy.

However, the existing threshold cryptography mainly focuses on how to afford secure data for users, few works consider another requirement for the cloud application that needs to protect the sensitive data. In order to protect the privacy of the information sent from the user to the cloud, our work follows the idea of Hada’s work and applies it to threshold signature setting. In this paper, we propose a secure obfuscation for encrypted threshold signature. The main contributions are as follows:

1. We propose an obfuscator that implements encrypted threshold signature (ETS) functionality, which can outsource the threshold signing rights of users to cloud server securely by obfuscation. Besides, this method can protect the sensitive leakage from the ETS program running on an untrusted sever.
2. We propose some security notions of ETS functionality and the corresponding obfuscator. Under the decisional linear assumption and computational Diffie–Hellman assumption, the proposed obfuscator satisfies the requirements of ACVBP and existentially unforgeability in the standard model.
3. We analyze the correctness of functionality preservation and polynomial slowdown. Meanwhile, the performance analysis of ETS functionality and the obfuscator are provided. Finally, we implement the proposed algorithms in a personal computer by using java pairing-based cryptography library.

The remainder of this paper is organized as follows. In section 2, we present some preliminaries including bilinear pairings, security problems and circuit obfuscators. In section 3, we present some build blocks will be used in our proposed schemes, then we propose an encrypted threshold signature scheme and the corresponding obfuscator based on linear encryption scheme and threshold signature. Section 4 analyzes the security and performance of our scheme from the perspectives of functionality preservation, ACVBP and existentially unforgeability. Section 5 presents our conclusion.

## Preliminary

### Bilinear pairings and security problems

In this section, we describe bilinear maps and hard problems [38]. Let consider two cyclic groups G and $G_{T}$ with the same prime order *q*, and let *g* is a generator of G. A bilinear map $\hat{e}:G\times G\to G_{T}$ need satisfy the following properties:

1. Bilinearity: For all g,h∈G, and $a,b\in Z_{q}$, $\hat{e}\left(g^{a},h^{b}\right)=\hat{e}{\left(g,h\right)}^{ab}$.
2. Non-degeneracy: There exists $a,b\in Z_{q}$, such that $\hat{e}\left(g^{a},g^{b}\right)\neq 1$.
3. Computability: For all g,h∈G, $\hat{e}\left(g,h\right)$ can be computed.

**Definition 1**. *The Decision Linear (DLLN) Problem is to decide whether a* + *b* = *c*, *given* $u,v,h,u^{a},v^{b},h^{c}\in G$ *for unknown* $a,b,c\in Z_{q}$. *The DLLN assumption states that, there is no PPT algorithm can solve the DLLN problem with non-negligible advantage*.

**Definition 2**. *The Computational Diffie-Hellman (CDH) Problem is that, given* $g,g^{x},g^{y}\in G$ *for unknown* $x,y\in Z_{q}$, *it is hard to compute g*^*xy*^. *The CDH assumption states that, there is no PPT algorithm can solve the CDH problem with non-negligible advantage*.

### Circuit obfuscators

In this section, we briefly review some notations of circuit obfuscators used in this paper [32]. We use $C={\left\{C_{\lambda }\right\}}_{\lambda \in N}$ to denote a class of probabilistic circles, here *C*_λ_ is the circuits in C of input length *l*_*in*_(λ). the notation *C* ← *C*_λ_ denotes the generation procedure. PPT denotes probability polynomial time. Obf denotes an obfuscator. poly(λ) indicates the set of all polynomials of λ. We now provide definitions of statistical difference and preserving functionality.

**Definition 3**. [32] *The statistical difference between C*_0_(*x*) *and C*_1_(*x*) *is given by*:

$$\begin{array}{c} △\left(C_{0}\left(x\right),C_{1}\left(x\right)\right)=\frac{1}{2}\sum_{y\in {\left\{0,1\right\}}^{l_{out}\left(\lambda \right)}}\left|\text{Pr}\left[o⟵C_{0}\left(x\right):o=y\right]-\text{Pr}\left[o⟵C_{1}\left(x\right):o=y\right]\right| \end{array}$$

**Definition 4**. *(Preserving Functionality)* [32] *A PPT machine Obf is a circuit obfuscator for a class of probabilistic circuits* $C={\left\{C_{\lambda }\right\}}_{\lambda \in N}$, *if for every probabilistic circuit C* ∈ *C*_λ_, *the following holds*:

$$\begin{array}{c} \text{Pr}[C^{\prime }⟵\text{Obf}\left(C\right):\forall x,△\left(C\left(x\right),C^{\prime }\left(x\right)\right)=0]=1. \end{array}$$

## Obfuscation of encrypted threshold signatures

Encrypted threshold signatures (ETS) functionality utilizes a threshold signature (TS) scheme, which was proposed in [21] and an asymmetric linear encryption scheme [39]. After that, we will give a detailed description of obfuscation.

### TS signature

The TS signature scheme is a tuple of algorithms ∏ = (Setup, Share-Sign, Share-Verify, Combine, Verify) such that:

- **Setup**(*params*, λ, *k*, *n*): Takes as input a security parameter λ∈N and a pair of integers (*k*, *n*) ∈ poly(λ), such that 1 ≤ *k* ≤ *n*, let $P=\{P_{1},P_{2},\ldots ,P_{n}\}$ denote a set of *n* participants (users).
  1. Choose system parameter $params=(G,G_{T},\hat{e},g,q)$.
  2. $g_{2},u^{\prime },U=\left(u_{1},u_{2},\cdots ,u_{n}\right)\in G^{n+2}$ are also generated by using GJKR’s DKG algorithm [22], respectively.
  3. To generate public key, *n* users jointly generate user public key *g*_1_ = *g*^*α*^ by using GJKR’s DKG.
  4. Each user *P*_*i*_ broadcasts *g*^*f*(*i*)^ for a random jointly generated degree *k* − 1 polynomial $f\in Z_{q}\left[X\right]$ such that *α* = *f*(0).
  5. User *P*_*i*_ gets the private key shares **SK** = (*sk*_1_, *sk*_2_, ⋯, *sk*_*n*_) as $sk_{i}=g_{2}^{f(i)}$ for *i* = 1 to *n*. Verification keys **VK** = (*vk*_1_, *vk*_2_, ⋯, *vk*_*n*_) as *vk*_*i*_ = *g*^*f*(*i*)^ for *i* = 1 to *n*.
  6. Output the public key *p* = (**VK**, *params*, *g*_1_, *g*_2_, *u*′, **U**), and each user is supplied with the private key share *sk*_*i*_.
- **Share-Sign**(*sk*_*i*_, *m*): To sign *m* = *m*_1_ *m*_2_ ⋯ *m*_*n*_ ∈ {0, 1}^*n*^, using *sk*_*i*_, choose $r_{i}\in Z_{q}$, compute the signature share $\sigma_{i}=\left(\sigma_{i1},\sigma_{i2}\right)=\left(sk_{i}{\left(u^{\prime }\prod_{i=1}^{n}u_{i}^{m_{i}}\right)}^{r_{i}},g^{r_{i}}\right).$
- **Share-Verify**(*p*, *m*, *i*, *σ*_*i*_): Given a signature share *σ*_*i*_, and the verification key *vk*_*i*_, the partial verification algorithm return 1 if $\hat{e}\left(\sigma_{i1},g\right)=e\left(g_{2},vk_{i}\right)\hat{e}\left(u^{\prime }\prod_{i=1}^{n}u_{i}^{m_{i}},\sigma_{i2}\right)$, else return 0.
- **Combine**(*p*, *m*, *i*, *σ*_*i*_)_*i*∈Φ_: For each *i* ∈ Φ, where a subset Φ ⊂ {1, 2, ⋯, *n*} and |Φ| = *k*. Let λ_*i*_ be the Lagrange coefficients so that $\alpha =f\left(0\right)=\sum_{i\in \Phi }\lambda_{i}f\left(i\right),$ compute the combined signature $\left(\bar{\sigma_{1}},\bar{\sigma_{2}}\right)=\left(\prod_{i\in \Phi }\sigma_{i1}^{\lambda_{i}},\prod_{i\in \Phi }\sigma_{i2}^{\lambda_{i}}\right).$
- **Verify** $(p,m,\bar{\sigma_{1}},\bar{\sigma_{2}}):$ Given signature $\left(\bar{\sigma_{1}},\bar{\sigma_{2}}\right)$, the receiver checks the equation $\hat{e}\left(\bar{\sigma_{1}},g\right)=\hat{e}\left(g_{2},g_{1}\right)\hat{e}\left(u^{\prime }\prod_{i=1}^{n}u_{i}^{m_{i}},\bar{\sigma_{2}}\right).$ If the equation holds, outputs 1, otherwise outputs 0.

### Linear encryption scheme

The linear encryption scheme consists of three algorithms ∑ = (Key generation algorithm(KG), Encrypt algorithm(Enc), Decrypt algorithm(Dec)), the algorithms are described as follows:

- **KG**(*params*): Parse system parameter $params=(G,G_{T},\hat{e},g,q)$, choose $a,b\in Z_{q}$ as the private key *sk*_*e*_, compute the encryption public key *pk*_*e*_ = (*pk*_*e*1_, *pk*_*e*2_) = (*g*^*a*^, *g*^*b*^).
- **Enc**(*m*, *pk*_*e*_): To encrypt message *m*, randomly choose $r,s\in Z_{q}$, compute
  $$\begin{array}{c} \tau =\left(\tau_{1},\tau_{2},\tau_{3}\right)=\left(pk_{e1}^{r},pk_{e2}^{s},g^{r+s}m\right). \end{array}$$
- **Dec**(*τ*, *sk*_*e*_): Given *τ* and *sk*_*e*_, compute $m=\frac{\tau_{3}}{\tau_{1}^{\frac{1}{a}}\tau_{2}^{\frac{1}{b}}}$.

Specifically, we denote the rerandomization algorithm by **ReRand**(*p*, *pk*_*e*_, (*τ*_1_, *τ*_2_, *τ*_3_)), which produces a new ciphertext $\left(\tau_{1}{\left(g^{a}\right)}^{r^{\prime }},\tau_{2}{\left(g^{b}\right)}^{s^{\prime }},\tau_{3}g^{r^{\prime }+s^{\prime }}\right)$, equivalent to the input ciphertext *τ*, under the public key *pk*_*e*_ = (*g*^*a*^, *g*^*b*^), using the additional random numbers *r*′ and *s*′.

### The ETS functionality

ETS functionality is composed of ETS.Setup, ETS.Sign, ETS.Verify. We give the concrete construction as follows:

- **ETS.Setup**(*params*, λ, *k*, *n*):
  1. Parse parameter $params=(G,G_{T},\hat{e},g,q)$.
  2. For users(participants), generate public keys and private shares by running (**VK**, *params*, *g*_1_, *g*_2_, *u*′, **U**, **SK**)← Setup(*params*, λ, *k*, *n*).
  3. For receiver(verifier), randomly choose $a,b\in Z_{q}$ as the receiver’s private key *sk*_*e*_, compute receiver’s public key *pk*_*e*_ = (*pk*_*e*1_, *pk*_*e*2_) = (*g*^*a*^, *g*^*b*^).
- **ETS.Sign**(**SK**, *m*, *p*, *pk*_*e*_): For *m* = *m*_1_ *m*_2_⋯*m*_*n*_ ∈ {0, 1}^*n*^, works as follows:
  1. Randomly choose $r_{j}\in Z_{q}$, and compute *σ*_*j*_←**Share-Sign**(*sk*_*j*_, *m*), that is
    $$\begin{array}{c} \sigma_{j}=\left(\sigma_{j1},\sigma_{j2}\right)=\left(sk_{j}{\left(u^{\prime }\prod_{i=1}^{n}u_{i}^{m_{i}}\right)}^{r_{j}},g^{r_{j}}\right). \end{array}$$
  2. Verify the validity of signature *σ*_*j*_ by
    $$\begin{array}{c} \hat{e}\left(\sigma_{j1},g\right)=\hat{e}\left(g_{2},vk_{j}\right)\hat{e}\left({\left(u^{\prime }\prod_{i=1}^{n}u_{i}^{m_{i}}\right)}^{r_{j}},\sigma_{j2}\right). \end{array}$$
  3. Compute the combined signature $\left(\bar{\sigma_{1}},\bar{\sigma_{2}}\right)$ ← **Combine**(*p*, *m*, *j*, *σ*_*j*_)_*j*∈Φ_, that is
    $$\begin{array}{c} \left(\bar{\sigma_{1}},\bar{\sigma_{2}}\right)=\left(\prod_{j\in \Phi }\sigma_{j1}^{\lambda_{j}},\prod_{j\in \Phi }\sigma_{j2}^{\lambda_{j}}\right) \end{array}$$
    for each *j* ∈ Φ, where a subset Φ ⊂ {1, 2, ⋯, *n*} and |Φ| = *k*. Let λ_*j*_ be the Lagrange coefficients so that $\alpha =f\left(0\right)=\sum_{j\in \Phi }\lambda_{j}f\left(j\right).$
  4. Randomly choose $x_{1},x_{2},y_{1},y_{2}\in Z_{q}$, encrypt $\left(\bar{\sigma_{1}},\bar{\sigma_{2}}\right)$ under the receiver’s public key *S*_1_← **Enc** $\left(pk_{e},\bar{\sigma_{1}}\right)$ and *S*_2_← **Enc** $(pk_{e},\bar{\sigma_{2}}),$ that is
    $$\begin{array}{ccc} S_{1} & = & \left(pk_{e1}^{x_{1}},pk_{e2}^{x_{2}},g^{x_{1}+x_{2}}\bar{\sigma_{1}}\right)\\ & = & \left(pk_{e1}^{x_{1}},pk_{e2}^{x_{2}},g^{x_{1}+x_{2}}\prod_{j\in \Phi }\sigma_{j1}^{\lambda_{j}}\right)\\ & = & \left(pk_{e1}^{x_{1}},pk_{e2}^{x_{2}},g^{x_{1}+x_{2}}\prod_{j\in \Phi }{\left(sk_{j}{\left(u^{\prime }\prod_{i=1}^{n}u_{i}^{m_{i}}\right)}^{r_{j}}\right)}^{\lambda_{j}}\right) \end{array}$$
    $$\begin{array}{ccc} S_{2} & = & \left(pk_{e1}^{y_{1}},pk_{e2}^{y_{2}},g^{y_{1}+y_{2}}\bar{\sigma_{2}}\right)\\ & = & (pk_{e1}^{y_{1}},pk_{e2}^{y_{2}},g^{y_{1}+y_{2}}\prod_{j\in \Phi }{\left(g^{r_{j}}\right)}^{\lambda_{j}}). \end{array}$$
  5. Output encrypted threshold siganture (*S*_1_, *S*_2_).
- **ETS.Verify**(*p*, *sk*_*e*_, *S*_1_, *S*_2_, *m*): Parse *p* = (**VK**, *params*, *g*_1_, *g*_2_, *u*′, **U**), and *m* = *m*_1_ *m*_2_⋯*m*_*n*_ ∈ {0, 1}^*n*^. Decrypt (*S*_1_, *S*_2_) to get $\left(\bar{\sigma_{1}},\bar{\sigma_{2}}\right)$, that is
  $$\begin{array}{ccc} \bar{\sigma_{1}} & = & \frac{g^{x_{1}+x_{2}}\prod_{j\in \Phi }{\left(sk_{j}{\left(u^{\prime }\prod_{i=1}^{n}u_{i}^{m_{i}}\right)}^{r_{j}}\right)}^{\lambda_{j}}}{pk_{e1}^{\frac{1}{a}x_{1}}pk_{e2}^{\frac{1}{b}x_{2}}}\\ & = & \prod_{j\in \Phi }{\left(sk_{j}{\left(u^{\prime }\prod_{i=1}^{n}u_{i}^{m_{i}}\right)}^{r_{j}}\right)}^{\lambda_{j}}. \end{array}$$
  and
  $$\begin{array}{ccc} \bar{\sigma_{2}} & = & \frac{g^{y_{1}+y_{2}}\prod_{j\in \Phi }{\left(g^{r_{j}}\right)}^{\lambda_{j}}}{pk_{e1}^{\frac{1}{a}y_{1}}pk_{e2}^{\frac{1}{b}y_{2}}}\\ & = & \prod_{j\in \Phi }{\left(g^{r_{j}}\right)}^{\lambda_{j}}. \end{array}$$
  then verify the encrypted signature by $\hat{e}\left(\bar{\sigma_{1}},g\right)=\hat{e}\left(g_{2},g_{1}\right)\hat{e}\left(u^{\prime }\prod_{i=1}^{n}u_{i}^{m_{i}},\bar{\sigma_{2}}\right),$ else return 0.

### The obfuscation of ETS functionality

From the description of the ETS functionality in above section, we regard a family of circuits $C_{ETS}={\left\{C_{\lambda }\right\}}_{\lambda \in N}$ for the ETS functionality, *C*_λ_ is a group of circuits $C_{p,\text{SK},pk_{e}}$. We can draw system parameters (**SK**, *pk*_*e*_, *p*) from $C_{p,\text{SK},pk_{e}}$. Given a circuit $C_{p,\text{SK},pk_{e}}$, the Obf_ETS_ works as follows:

- **Obf** ${}_{\text{ETS}}\left(C_{p,\text{SK},pk_{e}}\right):$
  1. Extract system parameters (*pk*_*e*_, **SK**, *p*).
  2. Parse parameter $params=(G,G_{T},\hat{e},g,q)$, **SK** = (*sk*_1_, *sk*_2_, ⋯, *sk*_*n*_) and **VK** = (*vk*_1_, *vk*_2_, ⋯, *vk*_*n*_).
  3. For each *j* ∈ {1, 2, ⋯, *n*}, randomly choose $x_{j1},x_{j2}\in Z_{q}$, encrypt user’s private share *sk*_*j*_ to run $\left(pk_{e1}^{x_{j1}},pk_{e2}^{x_{j2}},sk_{j}^{\prime }\right)\leftarrow \text{Enc}\left(pk_{e},sk_{j}\right)$, $sk_{j}^{\prime }=g^{x_{j1}+x_{j2}}sk_{j}$ is an encrypted form of the original signing key *sk*_*j*_, then compute $vk_{j}^{\prime }=g^{x_{j1}+x_{j2}}vk_{j}$. Suppose $t=\left(c_{j1},c_{j2},c_{j3}\right)=\left(pk_{e1}^{x_{j1}},pk_{e2}^{x_{j2}},sk_{j}^{\prime }\right)$.
  4. Construct an obfuscated circles *R*_*p*,*pke*,*t*_ that contains the values $(p,pk_{e},vk_{j}^{\prime },t).$
- *R*_*p*,*pke*,*t*_: The obfuscated circuit can be executed on any untrusted cloud server, and it does the following.
  1. On input security parameter λ, the circuit outputs (*pk*_*e*_, *p*).
  2. On input message *m* = *m*_1_ *m*_2_⋯*m*_*n*_ ∈ {0, 1}^*n*^, randomly choose $r_{j}^{\prime }\in Z_{q}$ to run $\sigma_{j}^{\prime }=\left(\sigma_{j1}^{\prime },\sigma_{j2}^{\prime }\right)$ ← **Share-Sign** $\left(sk_{j}^{\prime },m\right)$, that is
    $$\begin{array}{c} \sigma_{j1}^{\prime }=g^{x_{j1}+x_{j2}}sk_{j}{\left(u^{\prime }\prod_{i=1}^{n}u_{i}^{m_{i}}\right)}^{r_{j}^{\prime }}, \end{array}$$
    and
    $$\begin{array}{c} \sigma_{j2}^{\prime }=g^{r_{j}^{\prime }}. \end{array}$$
  3. Verify the validity of signature $\sigma_{j}^{\prime }$ by
    $$\begin{array}{c} \hat{e}\left(\sigma_{j1}^{\prime },g\right)=\hat{e}\left(g_{2},vk_{j}^{\prime }\right)\hat{e}\left(u^{\prime }\prod_{i=1}^{n}u_{i}^{m_{i}},\sigma_{j2}^{\prime }\right). \end{array}$$
  4. Compute the combined signature $\left(\bar{\sigma_{1}^{\prime }},\bar{\sigma_{2}^{\prime }}\right)$ ← **Combine** ${\left(p,m,\sigma_{j}^{\prime }\right)}_{j\in \Phi },$ that is
    $$\begin{array}{c} \left(\bar{\sigma_{1}^{\prime }},\bar{\sigma_{2}^{\prime }}\right)=\left(\prod_{j\in \Phi }\sigma_{j1}^{\prime \lambda_{j}^{\prime }},\prod_{j\in \Phi }\sigma_{j2}^{\prime \lambda_{j}^{\prime }}\right) \end{array}$$
    for each *j* ∈ Φ, where a subset Φ ⊂ {1, 2, ⋯, *n*} and |Φ| = *k*. Let $\lambda_{j}^{\prime }$ be the Lagrange coefficients so that $\alpha =f\left(0\right)=\sum_{j\in \Phi }\lambda_{j}^{\prime }f\left(j\right).$
  5. Compute $c_{1}=\prod_{j\in \Phi }{\left(c_{j1}\right)}^{\lambda_{j}^{\prime }}=pk_{e1}^{\sum_{j\in \Phi }\lambda_{j}^{\prime }x_{j1}},$ and $c_{2}=\prod_{j\in \Phi }{\left(c_{j2}\right)}^{\lambda_{j}^{\prime }}=pk_{e2}^{\sum_{j\in \Phi }\lambda_{j}^{\prime }x_{j2}}.$
  6. Randomly choose $x_{1}^{\prime },x_{2}^{\prime },y_{1}^{\prime },y_{2}^{\prime }\in Z_{q}$, rerandomize the generated signature ${\bar{\sigma_{1}}}^{\prime }$ by running $S_{1}^{*}$ ← **ReRand** $(p,pk_{e},\left(c_{1},c_{2},{\bar{\sigma_{1}}}^{\prime }\right)),$ that is
    $$\begin{array}{ccc} S_{1}^{*} & = & \left(S_{11}^{*},S_{12}^{*},S_{13}^{*}\right)\\ & = & \left(c_{1}pk_{e1}^{x_{1}^{\prime }},c_{2}pk_{e2}^{x_{2}^{\prime }},g^{x_{1}^{\prime }+x_{2}^{\prime }}\prod_{j\in \Phi }{\sigma_{j1}^{\prime }}^{\lambda_{j}^{\prime }}\right)\\ & = & \left(pk_{e1}^{\sum_{j\in \Phi }\lambda_{j}^{\prime }x_{j1}+x_{1}^{\prime }},pk_{e2}^{\sum_{j\in \Phi }\lambda_{j}^{\prime }x_{j2}+x_{2}^{\prime }},g^{x_{1}^{\prime }+x_{2}^{\prime }}\prod_{j\in \Phi }{\left(g^{x_{j1}+x_{j2}}sk_{j}{\left(u^{\prime }\prod_{i=1}^{n}u_{i}^{m_{i}}\right)}^{r_{j}^{\prime }}\right)}^{\lambda_{j}^{\prime }}\right)\\ & = & \left(pk_{e1}^{\sum_{j\in \Phi }\lambda_{j}^{\prime }x_{j1}+x_{1}^{\prime }},pk_{e2}^{\sum_{j\in \Phi }\lambda_{j}^{\prime }x_{j2}+x_{2}^{\prime }},g^{\sum_{j\in \Phi }\lambda_{j}^{\prime }\left(x_{j2}+x_{j1}\right)+x_{1}^{\prime }+x_{2}^{\prime }}\prod_{j\in \Phi }{\left(sk_{j}{\left(u^{\prime }\prod_{i=1}^{n}u_{i}^{m_{i}}\right)}^{r_{j}^{\prime }}\right)}^{\lambda_{j}^{\prime }}\right) \end{array}$$
    and run $S_{2}^{*}$ ← **Enc** $(pk_{e},{\bar{\sigma_{2}}}^{\prime }),$ that is
    $$\begin{array}{ccc} S_{2}^{*} & = & \left(S_{21}^{*},S_{22}^{*},S_{23}^{*}\right)\\ & = & \left(pk_{e1}^{y_{1}^{\prime }},pk_{e2}^{y_{2}^{\prime }},g^{y_{1}^{\prime }+y_{2}^{\prime }}\prod_{j\in \Phi }{\sigma_{j2}^{\prime }}^{\lambda_{j}^{\prime }}\right)\\ & = & (pk_{e1}^{y_{1}^{\prime }},pk_{e2}^{y_{2}^{\prime }},g^{y_{1}^{\prime }+y_{2}^{\prime }}\prod_{j\in \Phi }{\left(g^{r_{j}^{\prime }}\right)}^{\lambda_{j}^{\prime }}). \end{array}$$
  7. Output $(S_{1}^{*},S_{2}^{*}$).

Besides, the polynomial time property is evident as all the calculation here is valid in polynomial time. It is easily to verify that the obfuscated program by theorem 1.

**Theorem 1**. *The algorithm R*_*p*,*pke*,*t*_ *can pass verification*.

**Proof 1**. *For a valid ciphertext* $\left(S_{1}^{*},S_{2}^{*}\right)$, *receiver decrypts* $\left(S_{1}^{*},S_{2}^{*}\right)$, *the correctness of R*_*p*,*pke*,*t*_ *is elaborated as follows*:

$$\begin{array}{ccc} \bar{\sigma_{1}} & = & g^{\sum_{j\in \Phi }\lambda_{j}^{\prime }\left(x_{j2}+x_{j1}\right)+x_{1}^{\prime }+x_{2}^{\prime }}\prod_{j\in \Phi }{\left(sk_{j}{\left(u^{\prime }\prod_{i=1}^{n}u_{i}^{m_{i}}\right)}^{r_{j}^{\prime }}\right)}^{\lambda_{j}^{\prime }}/pk_{e1}^{\frac{1}{a}\sum_{j\in \Phi }\lambda_{j}^{\prime }x_{j1}+x_{1}^{\prime }}pk_{e2}^{\frac{1}{b}\sum_{j\in \Phi }\lambda_{j}^{\prime }x_{j2}+x_{2}^{\prime }}\\ & = & g^{\sum_{j\in \Phi }\lambda_{j}^{\prime }\left(x_{j2}+x_{j1}\right)+x_{1}^{\prime }+x_{2}^{\prime }}\prod_{j\in \Phi }{\left(sk_{j}{\left(u^{\prime }\prod_{i=1}^{n}u_{i}^{m_{i}}\right)}^{r_{j}^{\prime }}\right)}^{\lambda_{j}^{\prime }}/g^{\sum_{j\in \Phi }\lambda_{j}^{\prime }x_{j1}+x_{1}^{\prime }}g^{\sum_{j\in \Phi }\lambda_{j}^{\prime }x_{j2}+x_{2}^{\prime }}\\ & = & \prod_{j\in \Phi }{\left(sk_{j}{\left(u^{\prime }\prod_{i=1}^{n}u_{i}^{m_{i}}\right)}^{r_{j}^{\prime }}\right)}^{\lambda_{j}^{\prime }}. \end{array}$$

$$\begin{array}{ccc} {\bar{\sigma_{2}}}^{\prime } & = & g^{y_{1}^{\prime }+y_{2}^{\prime }}{\left(\prod_{j\in \Phi }g^{r_{j}^{\prime }}\right)}^{\lambda_{j}^{\prime }}∕{pk_{e1}^{y_{1}^{\prime }}}^{\frac{1}{a}}{pk_{e2}^{y_{1}^{\prime }}}^{\frac{1}{b}}\\ & = & g^{y_{1}^{\prime }+y_{2}^{\prime }}\prod_{j\in \Phi }{\left(g^{r_{j}^{\prime }}\right)}^{\lambda_{j}^{\prime }}∕g^{y_{1}^{\prime }}g^{y_{2}^{\prime }}\\ & = & \prod_{j\in \Phi }{\left(g^{r_{j}^{\prime }}\right)}^{\lambda_{j}^{\prime }} \end{array}$$

*The following equation shows that R*_*p*,*pke*,*t*_ *satisfies correctness*:

$$\begin{array}{ccc} \hat{e}\left(\bar{\sigma_{1}},g\right) & = & \hat{e}\left(\prod_{j\in \Phi }{\left(sk_{j}{\left(u^{\prime }\prod_{i=1}^{n}u_{i}^{m_{i}}\right)}^{r_{j}^{\prime }}\right)}^{\lambda_{j}^{\prime }},g\right)\\ & = & \hat{e}\left(\prod_{j\in \Phi }sk_{j}^{\lambda_{j}^{\prime }},g\right)\hat{e}\left(u^{\prime }\prod_{i=1}^{n}u_{i}^{m_{i}},\prod_{j\in \Phi }g^{r_{j}^{\prime }\lambda_{j}^{\prime }}\right)\\ & = & \hat{e}\left(\prod_{j\in \Phi }{\left(g_{2}^{f(j)}\right)}^{\lambda_{j}^{\prime }},g\right)\hat{e}\left(u^{\prime }\prod_{i=1}^{n}u_{i}^{m_{i}},\prod_{j\in \Phi }g^{r_{j}^{\prime }\lambda_{j}^{\prime }}\right)\\ & = & \hat{e}\left(g_{2},g^{\sum_{j\in \Phi }f\left(j\right)\lambda_{j}^{\prime }}\right)\hat{e}\left(u^{\prime }\prod_{i=1}^{n}u_{i}^{m_{i}},{\bar{\sigma_{2}}}^{\prime }\right)\\ & = & \hat{e}\left(g_{2},g^{\alpha }\right)\hat{e}\left(u^{\prime }\prod_{i=1}^{n}u_{i}^{m_{i}},{\bar{\sigma_{2}}}^{\prime }\right)\\ & = & \hat{e}\left(g_{2},g_{1}\right)\hat{e}\left(u^{\prime }\prod_{i=1}^{n}u_{i}^{m_{i}},{\bar{\sigma_{2}}}^{\prime }\right). \end{array}$$

## Security properties

In the threshold cryptosystem, we should consider a coalition of *k* curious but honest users attack against the proposed obfuscator. Therefore, we suppose that an adversary is capable of obtaining the private key shares of corrupted users against the obfuscator, excepting the user who generates the obfuscated implementation as a challenge, that is, an adversary can access the corruption oracle on any corrupted user, but corrupt up to *k* − 1 of the *n* players, the set of oracle restrictions dependent on *C* is defined as *R*(*C*). In this paper, we define *R*(*C*) = {*Corruption*, ∣Φ∣ ≤ *k* − 1}, which can be expressed as *Corruption*^∣Φ∣≤*k*−1^. Some security requirements of the proposed obfuscator are introduced in the following descriptions.

**Definition 5**. [34] *An obfuscator Obf for C meets the ACVBP w.r.t. dependent oracle set T*(*C*) *and restricted dependent oracle set R*(*C*) *if the following situation holds: There exists a PPT simulator S such that, for distinguisher D, arbitrary polynomial f, all sufficiently large* λ∈N, *and arbitrary z* ∈ {0, 1}^poly(λ)^,

$$\begin{array}{c} |\begin{array}{c} \text{Pr}[\begin{array}{cc} C⟵C_{\lambda }; & \\ C^{\prime }⟵\text{Obf}\left(C\right); & :b=1\\ b⟵D^{\ll C,T(C),R(C)\gg }\left(C^{\prime },z\right); \end{array}]\\ -\text{Pr}[\begin{array}{cc} C⟵C_{\lambda }; & \\ C^{\prime \prime }⟵S^{\ll C\gg }; & :b=1\\ b⟵D^{\ll C,T(C),R(C)\gg }\left(C^{\prime \prime },z\right); \end{array}] \end{array}|<\frac{1}{f(\lambda )}, \end{array}$$

*where D*^≪*C*,*T*(*C*),*R*(*C*)≫^ *means that D has sampling access to all oracles contained in T*(*C*) *and R*(*C*) *in addition to C*.

**Definition 6**. *Let* (*KG*, *Enc*, *Dec*) *and* (*Setup*, *Share* − *Sign*, *Share* − *Verify*, *Combine*, *Verify*) *be a couple of linear encryption and the threshold signature algorithms. The threshold algorithm is existentially unforgeable w.r.t. ETS functionality if the following situation has to be satisfied: There exists a PPT algorithm A, all sufficiently large* λ∈N, *arbitrary polynomial f, and arbitrary z* ∈ {0, 1}^poly(λ)^,

$$\begin{array}{c} |\text{Pr}[\begin{array}{c} \left(p,\text{SK}\right)⟵Setup\left(params,\lambda ,k,n\right),\left(sk_{e},pk_{e}\right)⟵KG\left(params\right);\\ \left(m,\sigma ,Q\right)⟵A^{\ll Share-Sign_{p,sk_{i}},Corruption^{|\Phi |\leq k-1}\gg }\left(p,pk_{e},z\right);\\ 1⟵Verify(m,\sigma ,p),m\notin Q; \end{array}]|<\frac{1}{f(\lambda )}, \end{array}$$

*where* $Share-Sign_{p,sk_{i}}$ *is the Share-Sign oracle, Corruption*^|Φ|≤*k*−1^ *is the corruption oracle such as no more than k* − 1 *private key shares can be obtained by adversary A in the whole game, Q is the set of message queried by A adaptively*.

**Definition 7**. *Let (KG, Enc, Dec) and (Setup, Share-Sign, Share-Verify, Combine, Verify) be a couple of linear encryption and the threshold signature algorithms. The threshold signature algorithm is existentially unforgeable w.r.t. the ETS Obfuscator if the following situation has to be satisfied: There exists a PPT algorithm A, all sufficiently large* λ∈N, *arbitrary polynomial f, and arbitrary z* ∈ {0, 1}^poly(λ)^,

$$\begin{array}{c} |\text{Pr}[\begin{array}{c} \left(p,\text{SK}\right)⟵Setup\left(params,\lambda ,k,n\right),\left(sk_{e},pk_{e}\right)⟵KG\left(params\right);\\ C^{\prime }⟵\text{Obf}\left(C\right);\\ \left(m,\sigma ,Q\right)⟵A^{\ll Share-Sign_{p,sk_{i}},Corruption^{|\Phi |\leq k-1}\gg }\left(p,pk_{e},z\right);\\ 1⟵Verify(m,\sigma ,p),m\notin Q; \end{array}]|<\frac{1}{f(\lambda )}, \end{array}$$

*where* $Share-Sign_{p,sk_{i}}$ *is the share sign oracle, Corruption*^|Φ|≤*k*−1^ *is the corruption oracle such as no more than k* − 1 *private key shares can be obtained by adversary A in the whole game, Q is the set of message queried by A adaptively*.

### Correctness

In this section, we identify the following goals that the obfuscator for ETS should satisfy.

1. Correctness: The correctness of an obfuscator requires “Preserving Functionality” as described in Definition 4.
2. Security: The obfuscator needs satisfy ACVBP with respect to *T*(*C*) and *R*(*C*) and existentially unforgeable with respect to ETS Obfuscator.

Below, we state the Theorem 2 which is a key result used to show the correctness of our construction.

**Theorem 2**. (Preserving Functionality) *The obfuscated program preserves the functionality of original ETS*.

**Proof 2**. *On receiving the encrypted threshold signature* (*S*_1_, *S*_2_), *that is*

$$\begin{array}{ccc} S_{1} & = & \left(pk_{e1}^{x_{1}},pk_{e2}^{x_{2}},g^{x_{1}+x_{2}}\prod_{j\in \Phi }{\left(sk_{j}{\left(u^{\prime }\prod_{i=1}^{n}u_{i}^{m_{i}}\right)}^{r_{j}}\right)}^{\lambda_{j}}\right) \end{array}$$

*and*

$$\begin{array}{ccc} S_{2} & = & \left(pk_{e1}^{y_{1}},pk_{e2}^{y_{2}},g^{y_{1}+y_{2}}\prod_{j\in \Phi }{\left(g^{r_{j}}\right)}^{\lambda_{j}}\right) \end{array}$$

*where* $x_{1},x_{2},y_{1},y_{2}\in Z_{q}.$

*On receiving the obfuscated program* $(S_{1}^{*},S_{2}^{*}),$ *that is*

$$\begin{array}{ccc} S_{1}^{*} & = & \left(pk_{e1}^{\sum_{j\in \Phi }\lambda_{j}^{\prime }x_{j1}+x_{1}^{\prime }},pk_{e2}^{\sum_{j\in \Phi }\lambda_{j}^{\prime }x_{j2}+x_{2}^{\prime }},g^{\sum_{j\in \Phi }\lambda_{j}^{\prime }\left(x_{j2}+x_{j1}\right)+x_{1}^{\prime }+x_{2}^{\prime }}\prod_{j\in \Phi }{\left(sk_{j}{\left(u^{\prime }\prod_{i=1}^{n}u_{i}^{m_{i}}\right)}^{r_{j}^{\prime }}\right)}^{\lambda_{j}^{\prime }}\right) \end{array}$$

*and*

$$\begin{array}{ccc} S_{2}^{*} & = & \left(pk_{e1}^{y_{1}^{\prime }},pk_{e2}^{y_{2}^{\prime }},g^{y_{1}^{\prime }+y_{2}^{\prime }}\prod_{j\in \Phi }{\left(g^{r_{j}^{\prime }}\right)}^{\lambda_{j}^{\prime }}\right) \end{array}$$

*where* $x_{1}^{\prime },x_{2}^{\prime },y_{1}^{\prime },y_{2}^{\prime },r_{j}^{\prime }\in Z_{q}.$

*We observe that both* (*S*_1_, *S*_2_) *and* $\left(S_{1}^{*},S_{2}^{*}\right)$ *are identically distributed*.

### Security proof

**Theorem 3**. *Under the DLLN assumption, the algorithm* Obf_ETS_ *is ACVBP with respect to dependent oracle* $T\left(C\right)=Share-Sign_{p,sk_{i}}$ *and restricted dependent oracle R*(*C*) = *Corruption*^∣Φ∣≤*k*−1^.

**Proof 3**. *Suppose* $C=C_{p,\text{SK},pk_{e}}$, $T\left(C\right)=Share-Sign_{p,sk_{i}}$ *and* *R*(*C*) = *Corruption*^∣Φ∣≤*k*−1^. *There are a pair of probabilities* (Pr_*Nick*_, Pr_*Junk*_) *that represent D*^≪*C*,*T*(*C*),*D*(*C*)≫^ *outputs 1, given the true and imitated distributions, respectively. We show that* **S** **K** = (*sk*_1_, *sk*_2_, ⋯, *sk*_*n*_) *and* $\bar{\text{Junk}}=\left(Junk_{1},Junk_{2},\cdots ,Junk_{n}\right)$ *are encrypted in the true and imitated distributions. Since the algorithm* Obf_ETS_ *is equivalent to the values* $\left(p,pk_{e},vk_{i}^{\prime },t\right)$. *So we can utilize a simulator S which imitates these values with sampling access to C*. *The values* (*p*, *pk*_*e*_) can be easily draw from *C*. In order to simulate $\left(t,vk_{i}^{\prime }\right)$. *Then S chooses n junk values and encrypts them using the receiver’s public encryption key pk*_*e*_.

*The detailed procedure of S is as below*.

1. *Using the sampling access to* $C_{p,\text{SK},pk_{e}}$ *to get* (*p*, *pk*_*e*_).
2. *Parse p* = (**V** **K**, *params*, *g*_1_, *g*_2_, *u*′, **U**), **V** **K** = (*vk*_1_, *vk*_2_, ⋯, *vk*_*n*_) and $params=(G,G_{T},\hat{e},g,q)$.
3. *Randomly choose* $Junk_{1},Junk_{2},\cdots ,Junk_{n}\in G$, and $\bar{x_{i1}},\bar{x_{i2}}\in Z_{q}$.
4. *Encrypt Junk*_*i*_ *using public key pk*_*e*_, $\left(c_{i1},c_{i2},c_{i3}\right)=\left(pk_{e1}^{\bar{x_{i1}}},pk_{e2}^{\bar{x_{i2}}},g^{\bar{x_{i1}}+\bar{x_{i2}}}Junk_{i}\right)$ *for i* = 1 *to n*.
5. *Compute* $\bar{vk_{i}}=g^{\bar{x_{i1}}+\bar{x_{i2}}}vk_{i}$ *for i* = 1 *to n*.
6. *Set Junk* = (*c*_*i*1_, *c*_*i*2_, *c*_*i*3_), *where i* = 1, 2, ⋯, *n*.
7. *Output* $\left(p,pk_{e},\bar{vk_{i}},Junk\right)$, *obviously*, $R_{p,pk_{e},Junk}$ *has the same distribution as* $R_{p,pk_{e},t}$.

*We will first prove that the output distributions of the simulator and the obfuscator are indistinguishable. We prove this by contradiction, assume that the probability that a distinguisher D*^≪*C*,*T*(*C*),*D*(*C*)≫^ *can distinguish between the probabilities described is not negligible. That is*, |Pr_*Nick*_ − Pr_*Junk*_| *is not negligible*.

$$\begin{array}{c} {\text{Pr}}_{Nick}=|\text{Pr}[\begin{array}{cc} \left(p,\text{SK}\right)⟵\text{Setup}\left(params,\lambda ,k,n\right),\left(sk_{e},pk_{e}\right)⟵\text{KG}\left(params\right); & \\ \left(c_{i1},c_{i2},c_{i3}\right)⟵\text{Enc}\left(pk_{e},sk_{i}\right),i=1,2,\cdots ,n; & \\ sk_{i}^{\prime }=c_{i3},i=1,2,\cdots ,n; & :b=1\\ b\leftarrow D^{\ll C_{p,\text{SK},pk_{e}},Share-Sign_{p,sk_{i}},Corruption^{|\Phi |\leq k-1}\gg }\left(p,pk_{e},sk_{i}^{\prime },z\right); \end{array}]| \end{array}$$

$$\begin{array}{c} {\text{Pr}}_{Junk}=|\text{Pr}[\begin{array}{cc} \left(p,\text{SK}\right)⟵\text{Setup}\left(params,\lambda ,k,n\right),\left(sk_{e},pk_{e}\right)⟵\text{KG}\left(params\right); & \\ \bar{\text{Junk}}=\left(Junk_{1},Junk_{2},\cdots ,Junk_{n}\right)\in G^{n}; & \\ \left(c_{i1},c_{i2},c_{i3}\right)⟵\text{Enc}\left(pk_{e},Junk_{i}\right),i=1,2,\cdots ,n; & \\ sk_{i}^{\prime }=c_{i3},i=1,2,\cdots ,n; & :b=1\\ b\leftarrow D^{\ll C_{p,\text{SK},pk_{e}},Share-Sign_{p,sk_{i}},Corruption^{|\Phi |\leq k-1}\gg }\left(p,pk_{e},sk_{i}^{\prime },z\right); \end{array}]| \end{array}$$

*Assume that the probability of D to win is not negligible, then we build a couple of adversaries* (*A*, *B*), *which attacks the semantic security of the encryption algorithm. First, A does as below*:

1. *Take as input* (*params*, *pk*_*e*_, *p*, *z*).
2. *Parse* $params=(G,G_{T},\hat{e},g,q)$.
3. *Generate the signers’ private key shares* **S** **K**.
4. *Parse* **S** **K** = (*sk*_1_, *sk*_2_, ⋯, *sk*_*n*_).
5. *Randomly choose* $Junk_{1},Junk_{2},\cdots ,Junk_{n}\in G$.
6. *Set m*_1_ = *sk*_*i*_ *and m*_2_ = *Junk*_*i*_.
7. *Output* (*m*_1_, *m*_2_, *pk*_*e*_).

*Given an encryption ciphertext ct* of *m*_*i*_, *the algorithm B can make a distinction between m*_1_ *and m*_2_ *by utilizing D*.

1. *Take as input* (*p*, *pk*_*e*_, *m*_1_, *m*_2_, *ct*, *z*).
2. *Parse* $params=(G,G_{T},\hat{e},g,q)$ *and ct*.
3. *Simulate* $D^{⪡C,T(C),D(C)\gg }\left(p,pk_{e},sk_{i}^{\prime },c_{i1},c_{i2},z\right)$.
4. *Output D’s output*.

*The advantage of attacker B is the same as the advantage of the distinguisher D to distinguish the output distributions of obfuscator and simulator. So if it’s not negligible, then it contradicts the DLLN assumption. Thus the advantage of D is negligible when given one tuple of ciphertexts, then the advantage when given three tuples is also negligible. So we conclude that the obfuscator satisfies ACVBP with dependent oracle set T*(*C*) *and restricted oracle setR*(*C*).

**Theorem 4**. *If* Obf_ETS_ *for ETS functionality is ACVBP w.r.t. dependent oracle* $T\left(C\right)=Share-Sign_{p,sk_{i}}$ *and restricted dependent oracle R*(*C*) = *Corruption*^∣Φ∣≤*k*−1^, *then the existentially unforgeable w.r.t. ETS functionality implies the existentially unforgeable w.r.t. ETS obfuscator*.

**Proof 4**. *The proof of this theorem is very similar to the proof in* [32], *see [103, Theorem 1], we thus omit the formal proof here*.

From Theorem 3 and Theorem 4, the TS scheme satisfies the existentially unforgeable, even if the adversary can obtain the obfuscated circuit. The obfuscator for ETS is mainly to enhance the security, and it is safe for the obfuscation circuit to be executed by any untrusted cloud server, and the cloud server could not get any useful information from it.

**Corollary 1**. *Under DLLN and CDH assumptions, TS scheme is existentially unforgeable w.r.t*. Obf_ETS_.

## Experimental results

### Theoretical performance analysis

Here we analyze the performance efficiency of our scheme, in terms of computational complexity when performing ETS.Sign, Obf_ETS_, *R*_*p*,*pke*,*t*_ and ETS.Verify operations. The result is showed in Table 1. In this table, Rand denotes the operation that randomly selects element, Add denotes addition, Mult denotes multiplication, Exp be an exponent operation, Inv denotes inverse operation. As shown in Table 1, the computational complexity of ETS.Sign and *R*_*p*,*pke*,*t*_ algorithms is linear in the number of *n* and *k*. All these operations are polynomial bounded operations and can be computed effectively. Therefore, all algorithms are efficient from a theoretical perspective.

**Table 1:** Computational overhead, where *n* is the number of users, *k* is the threshold number.

	Operation	ETS.Sign	Obf_ETS_	*R*_*p*,*pke*,*t*_	ETS.Verify
$Z_{q}$	Rand	*n* + 4	2*n*	*n* + 4	0
	Add	2	*n*	2*k*	0
	Inv	0	0	0	4
G	Mult	2*k* + *n* + 1	2*n*	4*k* + *n* + 1	0
	Exp	2*k* + 2*n* + 6	3*n*	4*k* + 2*n* + 6	4
	Inv	0	0	0	2
$G_{T}$	Mult	1	0	1	1
$\hat{e}:G\times G\to G_{T}$	Pair	3	0	3	3

### Implementation

To provide numerical results, we implement it to measure the performance of our scheme. Our implementation is written in *C* using the Pairing-Based Cryptography Library [40]. For the computations, we use the curve groups that are implemented in the Libpbc library. The computations are run on a PC with 3.70 GHz CPU frequency, and 4 GB of RAM. In the experiment, we use elliptical curves with a base field size of 512 bits and an embedding degree of 2. The security levels are selects as |*p*| = 512.

**Fig 1:**
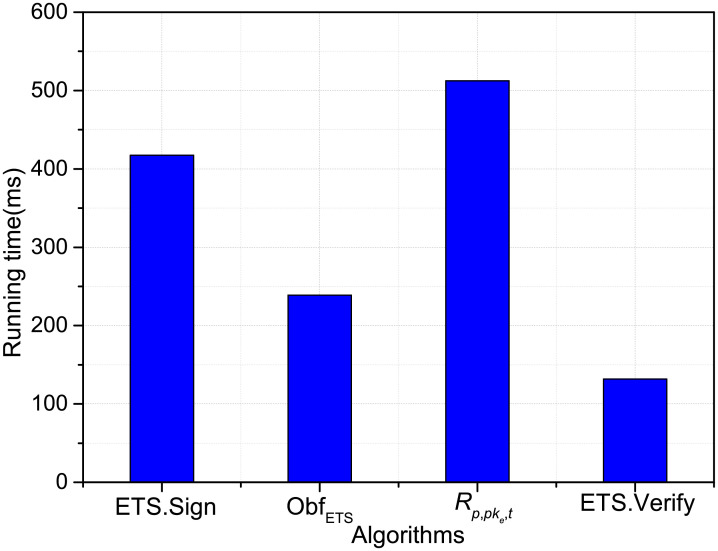
Execution time of the algorithms.

The following results denote the average running times of related cryptographic operations. In the experiment, the experimental result is the average number of 10 runs. We measure the running time of four algoritms, that is: ETS.Sign, Obf_ETS_, *R*_*p*,*pke*,*t*_ and ETS.Verify. The performing consequence of our scheme is provided in Fig 1 when *n* = 5 and *k* = 3. It is shown that the obfuscated implementation have high efficiency in general, because the algorithm needs perform more exponent operation.

**Fig 2:**
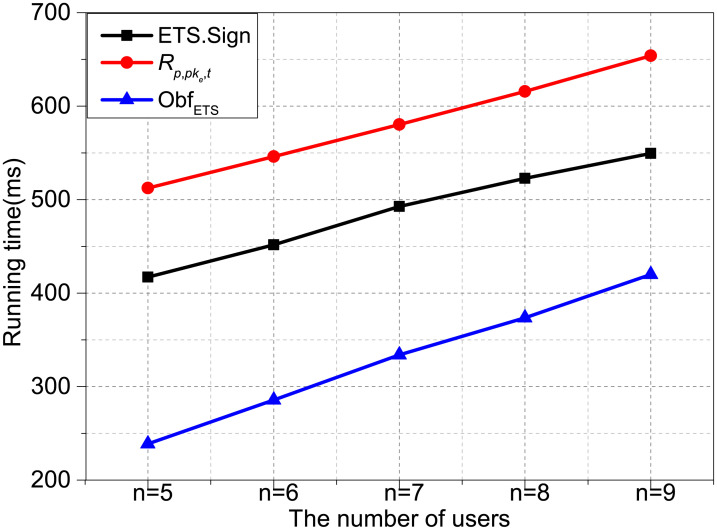
Time cost with k = 3.

**Fig 3:**
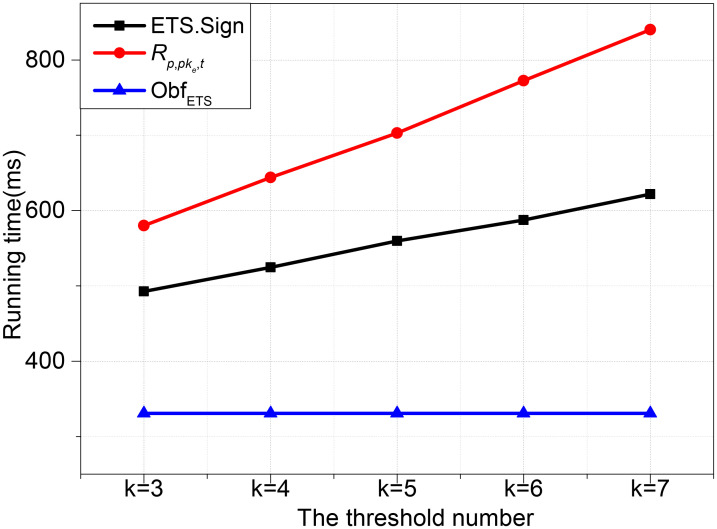
Time cost with n = 7.

Figs 2 and 3 show the time variety when the number of *n* and *k* as variables, respectively. Fig 2 shows the operations time of ETS.Sign, Obf_ETS_ and *R*_*p*,*pke*,*t*_ when *k* is set as 3 and the number of *n* is set varies from 5 to 9 increased by an interval of 1. Fig 3 shows the execution time of the three algorithms when *n* is set as 7 and the number of *k* is set varies from 3 to 7 increased by an interval of 1. We observe that *R*_*p*,*pke*,*t*_, ETS.Sign and Obf_ETS_’s time cost increases fastly along with the increasing of *n* and *k*. It can be seen from the results that *R*_*p*,*pke*,*t*_ is more costly than ETS.Sign with the same *n* or *k*.

## Conclusion

Obfuscation technique can provide much greater security for sensitive data from service providers in cloud computing. In this paper, we design an obfuscator for encrypted threshold signature, according to this technique, key shares are obfuscated before they are uploaded to the cloud services. In this regard, we can implement the program obfuscator run on a untrusted cloud sever, while hiding privacy-related sensitive information from the obfuscated program. The security analysis demonstrate that our scheme can meet the average case virtual black box property.

## Supporting information

S1 Fig Execution time of the algorithms. (DOC)

S2 Fig Time cost with k = 3. (DOC)

S3 Fig Time cost with n = 7. (DOC)

S1 Table Computational overhead, where n is the number of users, k is the threshold number. (DOC)
