# Supplementary figures and images for: Obfuscating encrypted threshold signature algorithm and its applications in cloud computing
Source: PLoS One. 2021 Apr 16;16(4):e0250259. doi: 10.1371/journal.pone.0250259 (PMC8051800; doi:10.1371/journal.pone.0250259)

S1 Fig. Execution time of the algorithms.


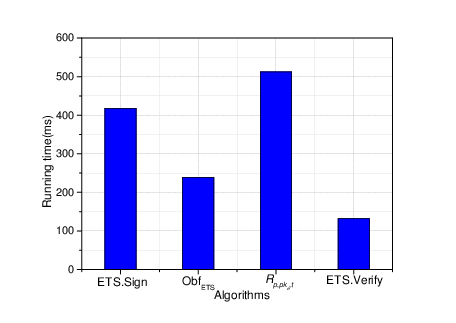

Supplement: S1 Fig — (DOC) [file pone.0250259.s001.doc]

S2 Fig. Time cost with k =3.


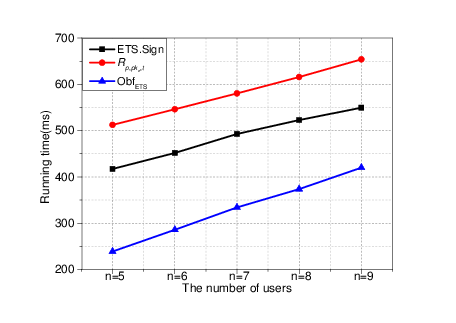

Supplement: S2 Fig — (DOC) [file pone.0250259.s002.doc]

S3 Fig. Time cost with n=7.


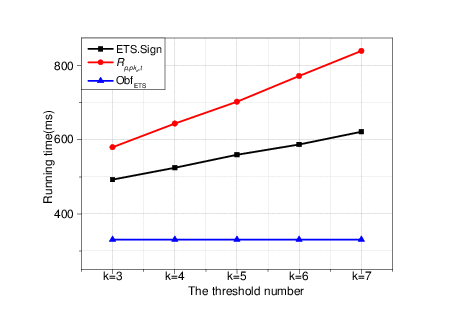

Supplement: S3 Fig — (DOC) [file pone.0250259.s003.doc]
