# Supplementary material for: Obfuscating encrypted threshold signature algorithm and its applications in cloud computing
Source: PLoS One. 2021 Apr 16;16(4):e0250259. doi: 10.1371/journal.pone.0250259 (PMC8051800; doi:10.1371/journal.pone.0250259)
Supplement: S1 Table — (DOC) [file pone.0250259.s004.doc]

S1 Table. Computational Overhead, where n is the number of uesrs, k is the threshold number.
